# Iatrogenic coronary artery fistula that developed during septal myectomy was detected intraoperatively through transesophageal echocardiography

**DOI:** 10.1186/s40981-024-00711-6

**Published:** 2024-04-24

**Authors:** Keita Uchiyama, Tsunehisa Tsubokawa

**Affiliations:** Department of Anesthesiology, The Jikei University School of Medicine, 3-25-8 Nishishimbashi, Minatoku, Tokyo 105-8461 Japan

To the Editor,

In the management of obstructive hypertrophic cardiomyopathy (HOCM), septal myectomy (the Morrow procedure) is an effective treatment for patients with persistent symptoms despite concurrent pharmacological treatment [1]. Coronary artery fistula is a complication of this procedure, which may develop in 19–23% [2, 3] of patients postoperatively. Although many fistulas close spontaneously, a subset of patients may remain undiagnosed, leading to significant complications such as myocardial ischemia or endocarditis, necessitating invasive intervention [2, 4]. Therefore, intraoperative detection and assessment for surgical repair of coronary fistulas are crucial. However, because the Morrow procedure is generally conducted using a transaortic approach, it is difficult for surgeons to detect fistula during operation. We present a case where transesophageal echocardiography (TEE) was instrumental in detecting a coronary artery fistula during terminal warm blood cardioplegia infusion.

A 50-year-old man (height: 171 cm, body weight: 82 kg, body mass index: 28.0 kg/m^2^) diagnosed with HOCM and experiencing exacerbating dyspnea was scheduled for the Morrow procedure. Preoperative transthoracic echocardiography (TTE) indicated a pressure gradient in the outflow tract of approximately 103 mmHg and moderate mitral regurgitation, with systolic anterior motion (SAM) of the mitral valve. Because of the extensive stenotic lesion in the outflow tract, the anterior wall approach was selected for the surgical procedure.

Post-induction TEE examination confirmed left ventricular outlet stenosis and SAM. Following the initiation of cardiopulmonary bypass (CPB), an incision was made in the left ventricular anterior wall, and hypertrophic myocardium resection was performed with care to preserve the His-Purkinje system. Upon the completion of resection and closure of the left ventricular wall, terminal warm blood cardioplegia was administered. Prior to the resumption of cardiac contractions, an abnormal flow within the left ventricular cavity emanating from the anterior wall was noted, prompting the diagnosis of a coronary artery fistula (Fig. 1). The fistula was well visualized, particularly in the mid-esophageal long-axis and transgastric basal short-axis views. The color Doppler wave exhibited a low-speed velocity of approximately 15 cm/s. The surgical team considered the possibility of fistula repair and decided to reassess post-CPB weaning. Upon discontinuation of CPB, TEE revealed the absence of shunt flow, indicating that repair was unnecessary. Follow-up TTE a week after the operation did not demonstrate the presence of the fistula.

**Fig. 1:**
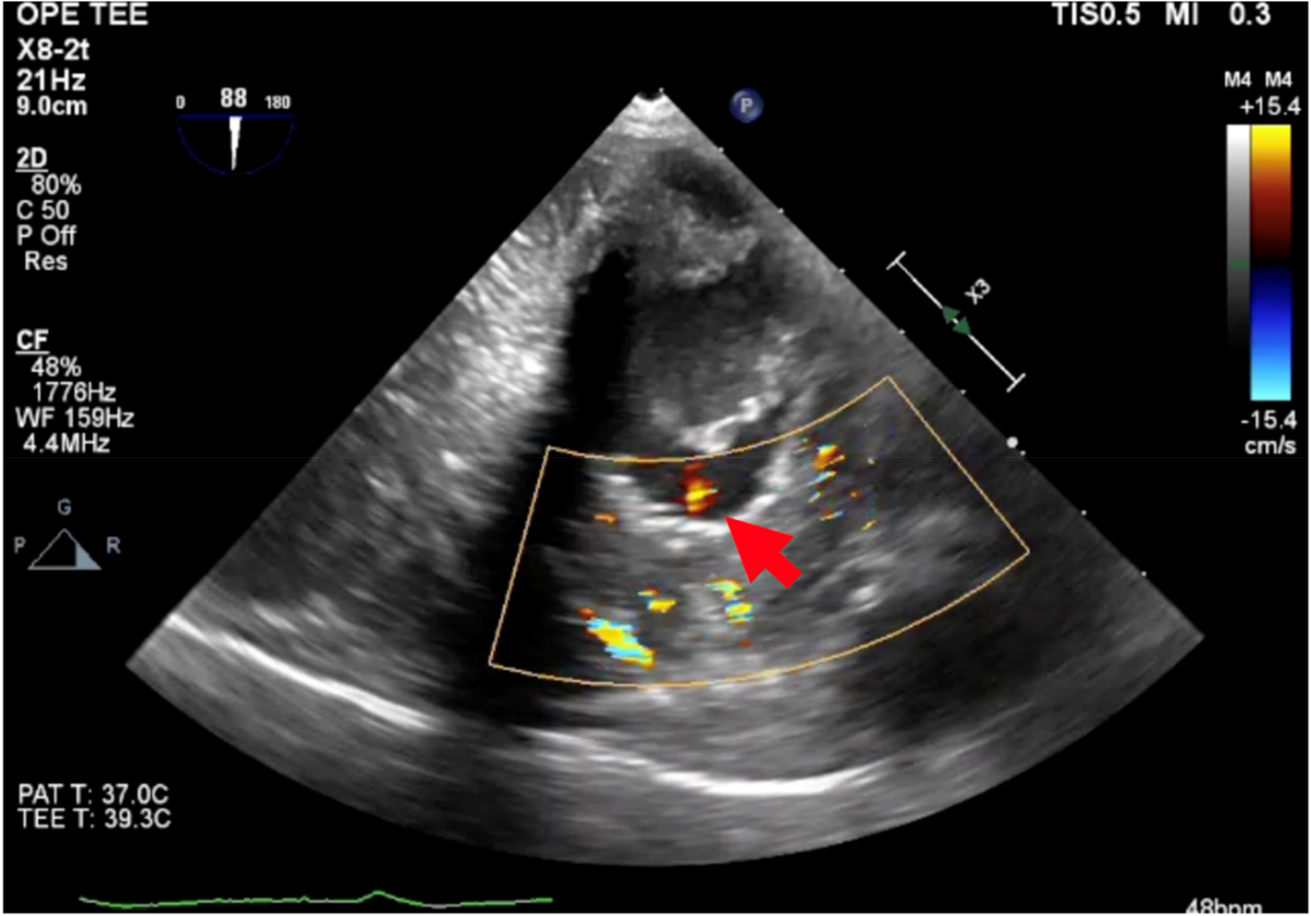
Transesophageal echocardiography during the infusion of terminal warm blood cardioplegia. A coronary artery fistula was detected at the anterior left ventricle wall by transesophageal echocardiography on the trans-gastric short-axis view

This case illustrates that coronary artery fistula may become apparent during terminal cardioplegia due to the significant pressure gradient between the coronary artery and collapsed left ventricle, which was approximately 35-mmHg injection pressure in this case. TEE is pivotal in detecting the coronary artery fistula intraoperatively prior to CPB weaning, thereby guiding the decision-making process regarding the necessity of fistula repair. Anesthesiologists should be vigilant during the window between the initiation of warm cardioplegia and resumption of cardiac activity for the detection of potential fistula induced by the Morrow procedure.

## Data Availability

Not applicable.

## Abbreviations

HOCM: Obstructive hypertrophic cardiomyopathy
TEE: Transesophageal echocardiography
TTE: Transthoracic echocardiography
SAM: Systolic anterior motion
CPB: Cardiopulmonary bypass
